# Simulation-based training for burr hole surgery instrument recognition

**DOI:** 10.1186/s12909-016-0669-2

**Published:** 2016-05-27

**Authors:** David B. Clarke, Nelofar Kureshi, Murray Hong, Maryam Sadeghi, Ryan C. N. D’Arcy

**Affiliations:** Division of Neurosurgery, QEII Health Sciences Centre, 1796 Summer Street, Halifax, NS B3H 3A7 Canada; Brain Repair Centre, Dalhousie University, 1348 Summer Street, Halifax, NS B3H 4R2 Canada; Simon Fraser University, Burnaby, BC V5A 1S6 Canada; Faculty of Applied Sciences, Simon Fraser University, 8888 University Drive, Burnaby, BC V5A 1S6 Canada

**Keywords:** Simulation, Education, Neurosurgery, Postgraduate surgical education

## Abstract

**Background:**

The use of simulation training in postgraduate medical education is an area of rapidly growing popularity and research. This study was designed to assess the impact of simulation training for instrument knowledge and recognition among neurosurgery residents.

**Methods:**

This was a randomized control trial of first year residents from neurosurgery residency training programs across Canada. Eighteen neurosurgery trainees were recruited to test two simulation-based applications: PeriopSim™ Instrument Trainer and PeriopSim™ for Burr Hole Surgery. The intervention was game-based simulation training for learning neurosurgical instruments and applying this knowledge to identify correct instruments during a simulated burr hole surgery procedure.

**Results:**

Participants showed significant overall improvement in total score (*p* < 0.0005), number of errors (*p* = 0.019) and time saved (*p* < 0.0005), over three testing sessions when using the PeriopSim™ Instrument Trainer. Participants demonstrated further performance-trained improvements when using PeriopSim™ Burr Hole Surgery.

**Conclusions:**

Training in the recognition and utilization of simulated surgical instruments by neurosurgery residents improved significantly with repetition when using PeriopSim™ Instrument Trainer and PeriopSim™ for Burr Hole Surgery.

## Background

Simulation in neurosurgical training has the potential to become an important educational tool for residents, surgeons, and perioperative clinicians [1]. Postgraduate medical education governing bodies including the Accreditation Council for Graduate Medical Education and the Royal College of Physicians and Surgeons of Canada mandate residency programs to teach and assess trainees in core competencies that encompass cognitive, psychomotor and affective domains [2, 3]. Traditionally, postgraduate medical learning has occurred through the clinical apprenticeship model; this model is likely to change to competency-based training [4] in which as core competency levels, also known as “milestones,” must be achieved and demonstrated by medical trainees before they progress as experts in their respective specialties. Restrictions in resident work hours, limitations in operative resources, and growing demands to improve the quality and safety of patient care are rapidly changing the surgical training environment. Given these limitations, it is becoming increasingly challenging to ensure that residents achieve the core competencies of establishing a clinical knowledge base, diagnosing medical disorders, and developing surgical technical skills.

Residency programs are recognizing that it is necessary to supplement the training of neurosurgical residents so that they can achieve required competencies. Simulation-based training and assessment is a valuable platform for teaching and evaluating procedural skill competence in many clinical disciplines [5, 6]. Training in a simulated environment provides a safe and risk-free setting for experiential learning and knowledge acquisition. Simulation-based tools are an effective means for learning and demonstrating competency in a variety of clinical skills [7–9].

Neurosurgical simulators complement traditional medical training. A recent review assessing simulation based technologies in neurosurgical resident education [10] found that sophisticated virtual reality (VR) simulators with haptic feedback and innovative 3D printing technology are popular methods of training in general, vascular and skull base neurosurgery. Validated simulators in spinal neurosurgery [11] include the ImmersiveTouch® (Immersive Touch, Inc., Chicago, Illinois) for percutaneous lumbar puncture, thoracic and lumbar pedicle screw placement, percutaneous spinal fixation, and vertebroplasty and the Dextroscope® (Volume Interactions Pte, Ltd., Singapore) for neuroanatomical instruction and preoperative planning.

**Fig. 1:**
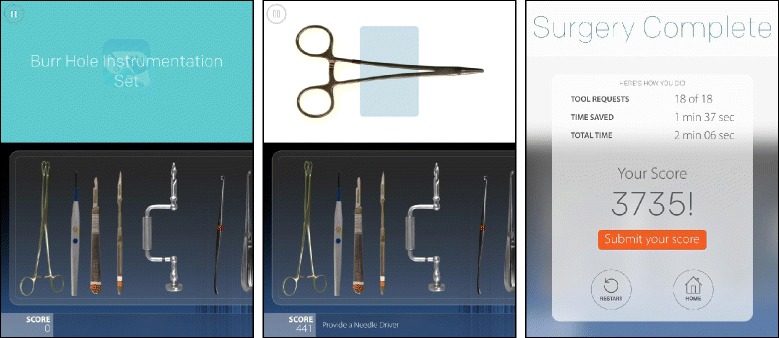
PeriopSim platform with burr hole instrumentation training and final score user screens

PeriopSim™ has been developed by Conquer Mobile in close collaboration with clinical surgeons and perioperative clinicians as an iPad application to provide a convenient user-friendly platform to facilitate learning about surgical procedures, instruments and techniques. It is a novel technology for surgery training, which in neurosurgery has two current modules: PeriopSim™ Instrument Trainer and PeriopSim™ for Burr Hole Surgery (Fig. 1). PeriopSim™ Instrument Trainer teaches general neurosurgical instruments using voice prompts and a simulated tool tray to guide the participant to select the correct tool. To create a realistic training environment, instruments displayed in the Instrument trainer are HD simulations of real surgery instruments. PeriopSim™ for Burr Hole Surgery teaches basic neurosurgical instrument usage for a burr hole surgical procedure. The burr hole surgery app is developed using a video clips of a real surgery. Using voice prompts, the interactive video of the burr hole procedure focuses on the use and appearance of instruments. Gamification techniques such as scoring and timed challenges motivate users to practice, sharpen skills and compete with colleagues [12, 13].

The PeriopSim™ platform was tested in a preliminary development study for residents at the Canadian Neurosurgery Rookie Camp. Rookie Camp provides an educational forum that emphasizes cognitive, behavioral and technical skills for all postgraduate year 1 (PGY-1) residents from various centers at the start of their training. This is national effort, in collaboration with the Neurosurgery Specialty Committee of the Royal College of Physicians and Surgeons of Canada and with support from the Canadian Neurosurgical Society (http://www.neurosurgeryrookie.ca) [14, 15].

The objective of this study was to evaluate whether PeriopSim™ Instrument Trainer and PeriopSim™ for Burr Hole Surgery improve the recognition of neurosurgical instruments.

## Methods

### Participants

The study involved a convenience sample of 18 PGY-1 neurosurgery trainees, drawn from 12 training programs participating in the 2014 Canadian Neurosurgery Rookie Camp in Halifax, Nova Scotia, Canada. Each study participant was provided with a unique QR barcode with an ID number to scan and enter the simulation applications. Participants were randomly divided into two groups (Group A, *n* = 10 and Group B, *n* = 8). Simple randomization based on QR codes was used to assign participants to study groups; even number QR codes were assigned to Group A and odd number QR codes were assigned to Group B. Participants were blinded to their group allocation. In addition, six experienced instructors (Group C, *n* = 6) performed specific simulation tasks.

### Design

**Fig. 2:**
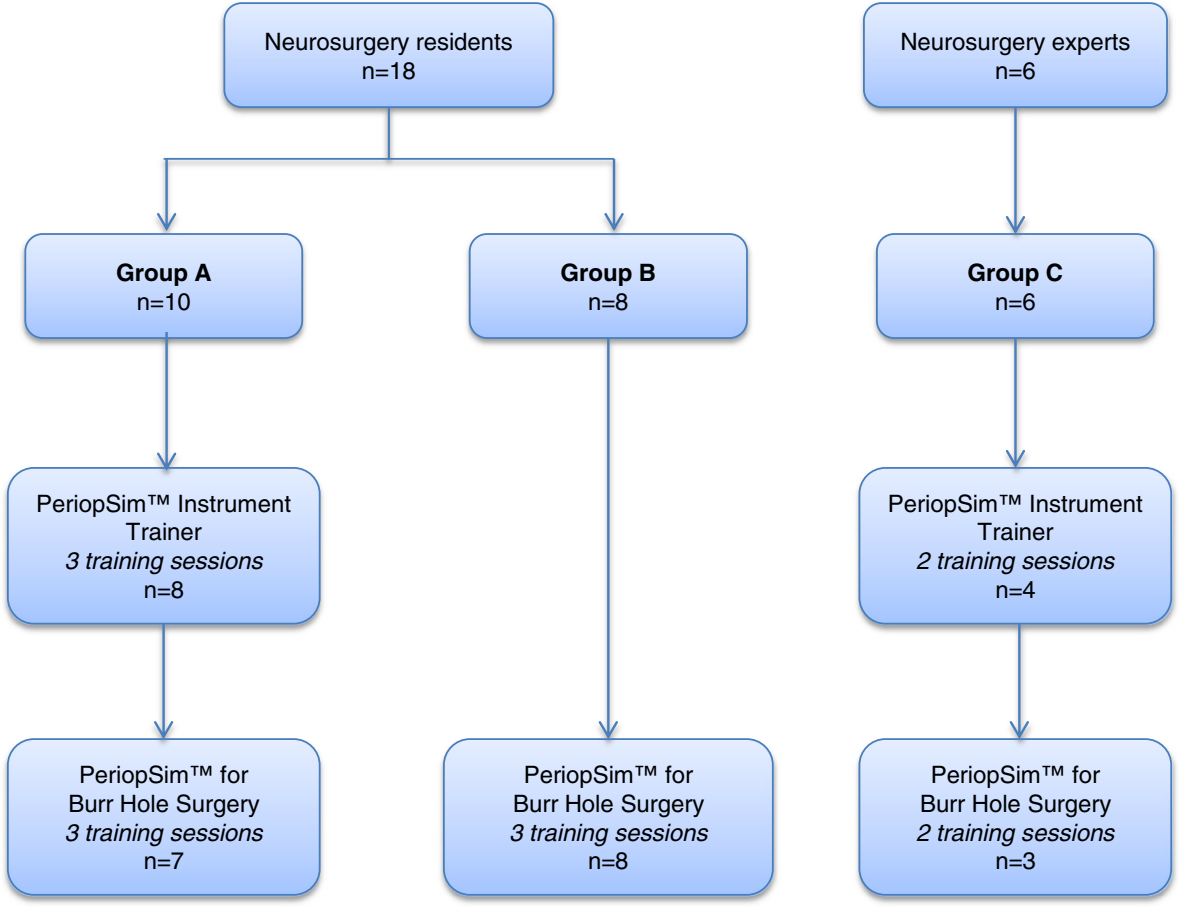
Research design methodology. Three participants in Group A arrived late to the testing stations and did not complete all tasks. Their data were excluded from analysis

The study was designed to assess whether the PeriopSim™ Instrument Trainer and PeriopSim™ for Burr Hole Surgery led to improvements in instrument knowledge and recognition with repetition. Participating residents were randomized into one of two groups. Figure 2 shows the experimental design for Group assignment across PeriopSim™ Instrument Trainer and PeriopSim™ for Burr Hole Surgery. Trainee randomization was performed to assess the effect of PeriopSim™ Instrument prior to participating in PeriopSim™ for Burr Hole Surgery. Trainees in Group A and Group B performed simulation tasks three times. Faculty experts at the Rookie Camp provided average recorded scores for PeriopSim™ Instrument Trainer and the PeriopSim™ for Burr Hole Surgery in order to establish expert-level performance baselines. Due to time constraints, experts (Group C) performed simulation tasks only twice. After each session, participants were presented with a dashboard displaying the performance outcomes and proficiency within the task (Fig. 1).

The primary outcome measures were time saved, number of errors, and total score. *Time saved* was defined as the duration in seconds from the time the participant correctly submitted an instrument prior to the maximum allotted time. *Number of errors* was counted as the number of incorrectly selected instruments on the first attempt. Any additional attempts were discarded, but participants were required to select the correct instrument in order to move on. The algorithm for *total score* was gamification-based and was dependent upon the number of correct responses in the first attempt and time saved. To determine the total score, the system used a multiplication factor of 200 per second of time saved and a multiplication factor of 100 for the number of correctly identified instruments on the first attempt. The algorithm for total score was created by the simulation development team and the formula was weighted towards time saved to encourage users to anticipate correct instruments.

### Statistical analysis

Performance measures (total score, number of errors and time saved) were reported as mean and standard error of the mean (SEM) for all groups. An omnibus repeated-measure ANOVA was conducted on performance metrics to evaluate significant change over testing sessions (using conservative degrees of freedom to adjust for potential alpha inflation). Post-hoc t-test analyses were then used to evaluate specific differences in the total score, number of errors, and time saved of subjects belonging to all three groups. The significance level was set at *p* < 0.05. All statistical analyses were performed using SPSS version 21 (IBM Corp., Armonk, NY, USA).

## Results

### PeriopSim™ instrument trainer

**Fig. 3:**
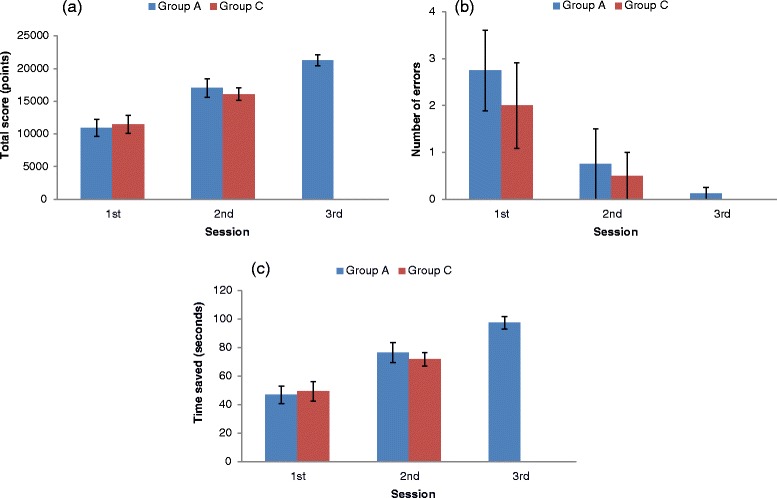
Performance measures (**a**) total score (**b**) number of instrument errors, and (**c**) time saved for *Group A* (residents) and *Group C* (experts) using PeriopSim™ Instrument Trainer. Data are given as means and standard error of the means

Figure 3 shows performance gains for Group A (compared with Group C) over three sessions of using PeriopSim™ Instrument Trainer. Repeated-measures ANOVA demonstrated a main effect of total scores, with a significant difference across the three training sessions (*p* < 0.0005). Post hoc tests (Bonferroni corrected) revealed that the total score on the PeriopSim™ Instrument Trainer increased significantly from session 1 to session 2 (10937 ± 1289 vs. 17029 ± 1427, respectively, *p* = 0.008). By session 3, Group A residents achieved a mean score of 21264 ± 873, which was significantly different from session 1 (*p* < 0.0005) and session 2 (*p* = 0.002) total scores (Fig. 3a).

Number of errors also differed significantly between three training sessions for Group A (*p* =0.013; Fig. 3b). Errors in identification of surgical instruments decreased from session 1 to session 2 (2.8 ± 0.9 vs. 0.8 ± 0.8), but this change was not statistically significant (*p* = 0.189). There was a significant drop in the number of errors between session 1 and session 3 (*p* = 0.046).

Group A residents saved time in the testing sessions by anticipating instruments while using the PeriopSim™ Instrument Trainer (Fig. 3c). There were additional savings in time with each repetition of the simulation: between session 1 and session 2 (*p* = 0.007) as well as between session 2 and session 3 (*p* = 0.002). By the third session, residents had doubled their saved time during simulation compared with their initial session.

The results demonstrated that the score, number of errors and time saved for Group C was not significantly different between the two testing sessions. As Group C was used to establish expert level performance, we also compared Group A’s performance to Group C. By the second session, Group A’s total score, number of errors, and time saved were not different from Group C.

### PeriopSim™ for burr hole surgery

**Fig. 4:**
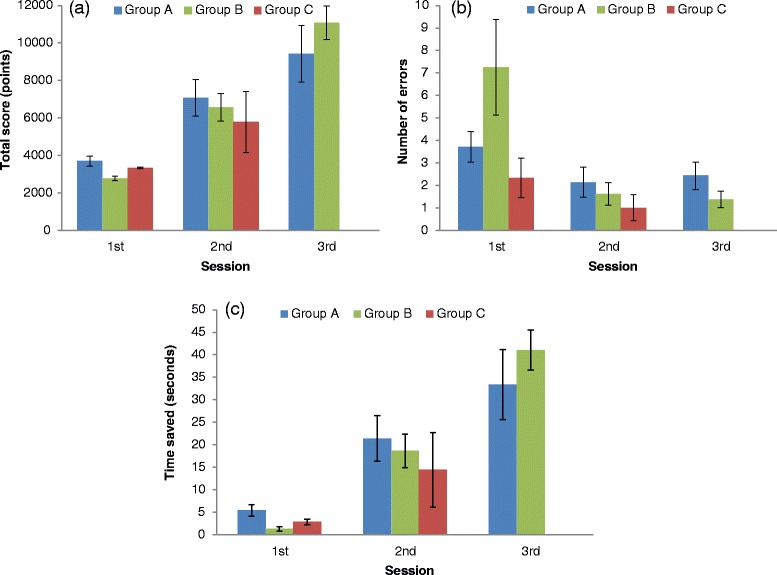
Performance measures (**a**), total score (**b**), number of instrument errors, and (**c**) time saved for all groups using PeriopSim™ for Burr Hole Surgery. Data are given as means and standard error of the mean

Group A and Group B performed PeriopSim™ in a simulated Burr Hole Surgery over three consecutive sessions (Fig. 2). Figure 4 shows how the groups performed.

In Group A, PeriopSim™ for Burr Hole Surgery total scores showed a significant increase between session 1 and session 2 (3703 ± 269 vs. 7062 ± 975, *p* = 0.037), but not between session 2 and session 3 (*p* = 0.136; Fig. 4a). The decrease in the number of errors for Group A was not significantly different between session 1 and session 2 (3.7 ± 0.7 vs. 2.1 ± 0.7, *p* = 0.548) or between session 2 and session 3 (*p* = 0.815; Fig. 4b).

Group A showed an increase in the time saved from session 1 to session 3 (5.4 ± 1 vs. 33.4 ± 8 sec.), which was statistically significant (*p* = 0.032). However, the difference in time saved was not significant from session 1 to session 2 (*p* = 0.055) or session 2 to session 3 (*p* = 0.156; Fig. 4c).

In Group B, PeriopSim™ for Burr Hole Surgery showed similar results to that of Group A. There was a significant increase in total scores between session 1 and session 2 performance (2767 ± 123 vs. 6565 ± 724, *p* = 0.002) and also between session 2 and session 3 (*p* = 0.005; Fig. 4a). The decrease in the number of errors was not significantly different over the three sessions (Fig. 4b).

As was seen in Group A, Group B demonstrated significant additional savings in time with each repetition of the simulated surgery. Time saved from session 1 to session 2 (1.3 ± 0.5 vs. 18.7 ± 3.7 sec.) was statistically significant (*p* = 0.005) and time saved from session 2 to session 3 was also significant (*p* = 0.005).

We compared the performance of trainees from Group B to those of Group A who had performed PeriopSim™ Instrument Trainer prior to PeriopSim™ for Burr Hole Surgery. Although the absolute numbers of errors were greater in Group B during session 1 (Fig. 4b), this difference was not statistically significant. With the exception of total score (*p* = 0.006) and time saved in session 1 (*p* = 0.008), there were no significant differences in performance between Group A and Group B.

As one might expect, Group C’s expert performance in PeriopSim™ for Burr Hole Surgery was not different over the testing sessions. By session 2, the mean score, number of errors, and time saved was not significantly different between the non-experts (Groups A and B) versus the experts (Group C).

## Discussion

Results from the experiment support the hypothesis that recognition of surgical instruments by residents improves with repetition when using the PeriopSim™ platform. Trainees from Group A, who performed PeriopSim™ Instrument Trainer, showed a significant increase in total score and time saved and a decrease in the number of errors over three testing sessions. Trainees with and without prior training on the instrument trainer displayed total score and time saved improvement over repeated sessions on PeriopSim™ for Burr Hole Surgery. Furthermore, there was a tendency to have fewer errors in performing the Burr Hole Surgery if there was prior training on the Instrument Trainer, but this was not statistically significant.

Residents demonstrated an improvement in overall performance using the PeriopSim™ Instrument Trainer, yet their performance on burr hole surgery simulation was not better than residents in Group B, who had no prior instrument training. This finding suggests that experience with the Instrument Trainer does not translate into a simulated operating room (OR) experience; however, the instruments used in the simulated burr hole surgery are quite basic and the residents may have had familiarity with them based on previous neurosurgical OR experience (something we did not assess).

Previous studies have established that simulation-based training can be effectively implemented in neurosurgical residency programs to complement the traditional apprenticeship model [14, 16]. Operational definitions of performance metrics of simulation training are ambiguous; however, there is consensus that metric units should capture accuracy, errors and efficiency of procedural steps [17]. This study evaluated the PeriopSim™ platform as a method of simulation-based training of surgical instruments in a controlled environment. Performance metrics of trainees showed progressive improvement over time, indicating that the learning curve for instrument recognition during a simulated burr hole surgery may be shortened with this approach.

Limitations of this study include a small sample size and lack of construct validity; however, the findings provide a useful baseline for assessing the differences in instrument recognition that may be found between postgraduate trainees who perform various simulation tasks. Larger studies are required to validate the effect of iPad-based simulation training of neurosurgical instruments. Our results may be influenced by confounding factors such as prior neurosurgical experience of postgraduate trainees; this was not assessed in the present study and may be a source of potential bias. Other factors such as age, gender, and handedness may also potentially influence outcomes, but were not assessed. Importantly, this study did not measure whether trainees were able to retain knowledge learned from simulation-based training after the completion of Rookie Camp, another key area of further investigation.

Postgraduate medical educational programs are increasingly examining the potential role of simulation-based training as a cost-effective and efficient tool in surgical education. Surgical simulation offers residents opportunities to practice and become competent in procedural skills without exposing patients to unnecessary risk [1]. The use of interactive game-based learning is an emerging technology, which can improve residents’ motivation and engagement in simulation-based learning. Future work will involve evaluating the transference of skills from simulation training to the real environment of the neurosurgical operating room.

Simulation training is becoming increasingly popular in many surgical disciplines [18, 19] and is rapidly emerging as an alternate modality to augment traditional surgical training. Findings from this study demonstrate that recognition for burr hole surgery instruments can be improved with repetitive simulation training using the PeriopSim platform. Although not examined in this study, this simple yet effective technology can be deployed internationally and would prove beneficial for novice surgeons and other surgical team members in any healthcare system.

## Conclusion

PeriopSim™ is a computer-based simulation that can effectively improve performance of surgical instrument recognition. Future work and further simulation development are required to evaluate this type of novel technology as an effective educational tool for the members of the surgical team.

## Abbreviations

OR: operating room
PGY: postgraduate year
SEM: standard error of mean
